# Bidirectional regulation between ferroptosis and tumor-associated macrophages: mechanisms, strategies, and therapeutic perspectives

**DOI:** 10.3389/fonc.2026.1905711

**Published:** 2026-08-13

**Authors:** Xiaoping Yu, Huiling Li, Siye Liu, Zhaodong Ai, Lanlan Wang, Lian Jian

**Affiliations:** Department of Diagnostic Radiology, Hunan Cancer Hospital and the Affiliated Cancer Hospital of Xiangya School of Medicine, Central South University, Changsha, China

**Keywords:** cGAS–STING, ferroptosis, immune regulation, iron metabolism, lipid peroxidation, tumor microenvironment, tumor-associated macrophages

## Abstract

Ferroptosis arises when iron-dependent phospholipid peroxidation overwhelms the system xc−–glutathione–GPX4 axis and parallel antioxidant defenses. Within tumors, however, the immune consequence of this death process depends on how iron and oxidized material move between malignant and immune compartments. Tumor-associated macrophages (TAMs) occupy this interface because they recycle iron, engulf dying cells and oxidized membranes, and modify the redox state of neighboring cells. These activities may confine injury to tumor cells, propagate lipid damage into immune cells, or protect malignant cells through vesicle cargo and antioxidant exchange. Ferroptotic death combines loss of viability with iron-dependent phospholipid peroxidation attenuated by an appropriate inhibitor, with genetic corroboration where feasible. Tumor-restricted ferroptosis can support antigen handling while macrophage and dendritic-cell functions remain intact. As oxidized material exceeds local clearance and antioxidant capacity, the same insult can impair CD8+ T cells and phagocytic macrophages and favor immune suppression. Three linked modules organize this process: iron-flux redistribution, lipid-hydroperoxide spectra and receptor sensing, and phagocytic clearance and antioxidant buffering. Iron flux identifies the first injured compartment, lipid species and receptor distribution shape the ensuing immune response, and clearance determines the duration and spread of injury. The framework thereby explains how TAM state, tumor niche, and treatment schedule redirect a common oxidative insult without proposing an additional ferroptosis execution pathway. Clinical translation will require ferroptosis to remain concentrated in the intended compartment while CD8+ T cells and homeostatic phagocytes retain function. Bulk iron, oxidative markers, and transcriptomic signatures provide complementary signals but cannot resolve the injured cell population.

## Introduction

Immune checkpoint inhibitors (ICIs) targeting cytotoxic T-lymphocyte-associated protein 4 (CTLA-4), programmed cell death protein 1 (PD-1)/programmed death-ligand 1 (PD-L1), and lymphocyte activation gene 3 (LAG-3) have produced durable responses in several malignancies (1–3). However, many immune-excluded or myeloid-rich tumors remain resistant. In these tumors, tumor-associated macrophages (TAMs) comprise states defined by ontogeny, transcriptional program, and spatial location, with phagocytic, angiogenic, matrix-remodeling, and immunoregulatory functions that vary with tissue of origin, niche, and treatment exposure (4, 5). Ferroptosis was initially defined as an iron-dependent, non-apoptotic death program and is now understood as lethal phospholipid peroxidation constrained by the system xc−–GSH–GPX4 axis and parallel antioxidant defenses (6–8). TAMs connect these processes by regulating the substrates available for lipid peroxidation and the fate of ferroptotic material. Oxidized lipids, damage-associated molecular patterns (DAMPs), extracellular iron, and reactive oxygen species (ROS) may accompany ferroptotic death, although none is specific to it (9, 10). This distinction is particularly relevant to macrophages, which clear senescent erythrocytes, recycle iron, and adapt iron uptake, storage, and export to local inflammatory signals (11, 12).

**Figure 1:**
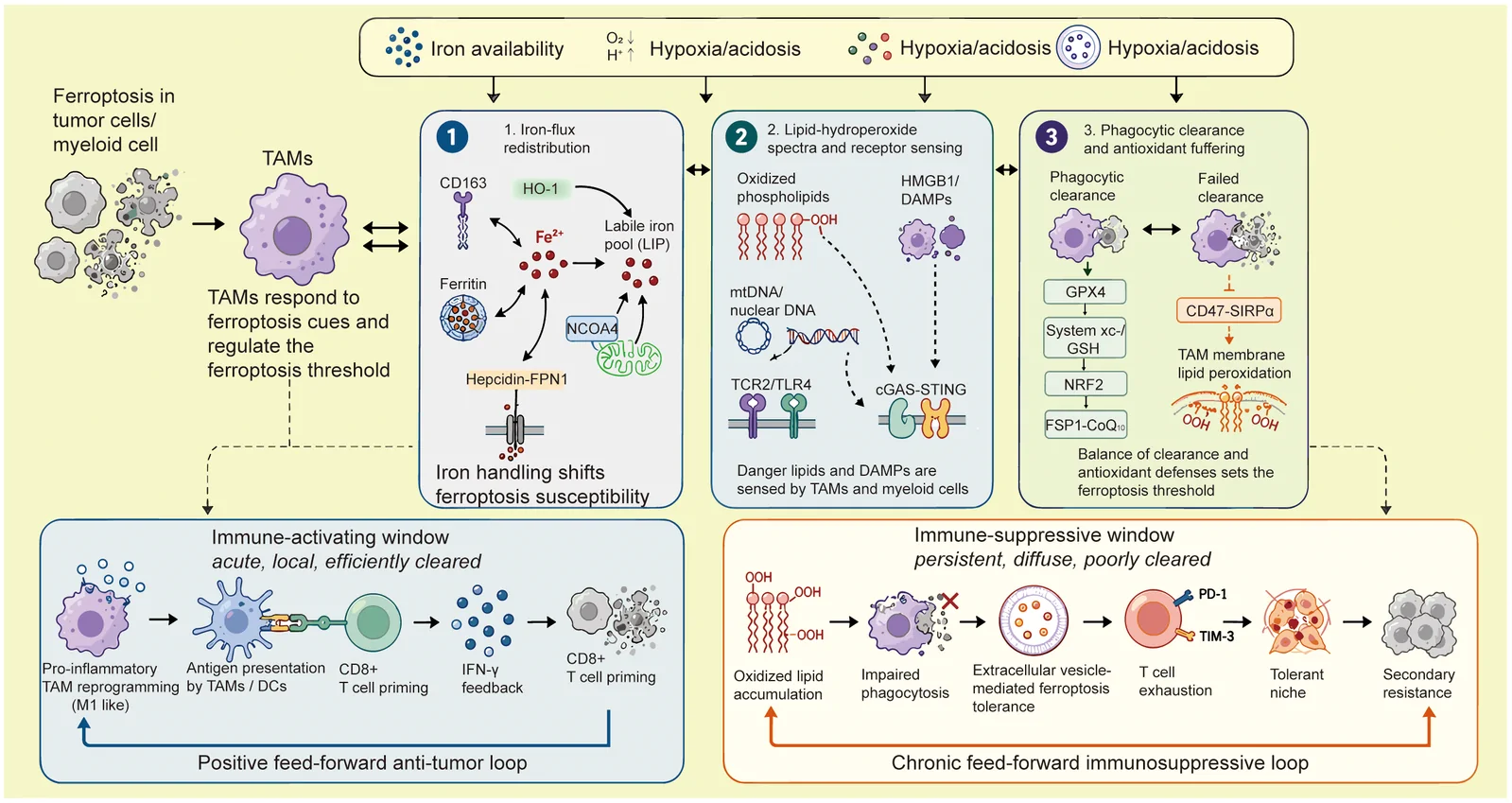
The bidirectional ferroptosis–TAM axis in tumor immunity. This schematic organizes the ferroptosis–TAM axis into three modules. Module 1 covers iron-flux redistribution through CD163/HO-1 heme handling, ferritin, NCOA4-linked ferritinophagy, the LIP, and the hepcidin–FPN1 checkpoint. Module 2 covers oxidized phospholipids, DAMPs, nucleic-acid stress, receptor sensing, and cGAS–STING. Module 3 covers phagocytic clearance and GPX4, system xc−–GSH, NRF2, and FSP1–CoQ10 buffering. Macrophage iron recycling and antioxidant defense are shown as core cellular processes. Each ligand–receptor or clearance route follows the ligand, receptor-bearing cell, tumor system, and exposure duration described in the text. An early, spatially restricted event with intact clearance is depicted as activation-permissive. Persistent or diffuse peroxidation that exceeds clearance or injures immune cells is depicted as suppression-permissive. These configurations occupy opposite ends of increasing tissue spread. Arrow direction represents the movement of iron, oxidized material, or cellular signals.

In preclinical tumor models, ferroptosis and TAMs form a bidirectional relationship whose direction changes with the identity of the dying cell, local myeloid state, spatial exposure, dose, and treatment timing. On the tumor-to-macrophage arm, ferroptosis-related perturbations alter macrophage metabolism and inflammatory state, which can modify tumor-cell survival (13, 14). On the reciprocal arm, TAM-derived extracellular vesicles and other forms of intercellular exchange shift the ferroptosis threshold of recipient tumor cells (15–17). The three-module framework follows the material transferred across these arms (**Figure 1**). Iron-flux redistribution identifies the cell in which the labile iron pool rises. The resulting lipid-hydroperoxide spectrum and receptor distribution shape the response of nearby immune cells. Phagocytic clearance and antioxidant buffering then govern how long oxidized material remains in tissue. Stable-isotope iron tracing after hepcidin–ferroportin perturbation assesses the first module. Lipidomics combined with receptor blockade tests the second, and efferocytosis coupled to cell-resolved antioxidant readouts evaluates the third. Because phagocytosis transfers iron and oxidized membranes into macrophages, the clearance module feeds back into iron storage and lipid sensing. Dose and sequence can consequently redirect the same tumor-cell injury toward local clearance, immune activation, or broader immune-cell damage.

Unlike cell-autonomous ferroptosis maps, the three modules follow injury across tumor and immune compartments. Iron flux identifies the first cell to acquire a labile iron burden compatible with ferroptosis. Oxidized phospholipid composition and receptor distribution shape the response in recipient cells, while phagocytic clearance and antioxidant buffering govern whether this material remains local or accumulates across the tumor microenvironment (TME) (18). When debris production remains within myeloid uptake and redox capacity, antigens and danger signals can be processed locally. Once production exceeds that capacity, oxidized material persists and reaches CD8+ T cells, dendritic cells, or clearance-competent macrophages, weakening antitumor immunity (18). Ferroptotic death combines loss of viability with iron-dependent phospholipid peroxidation that is attenuated by an appropriate ferroptosis inhibitor (6, 8, 10). Concordant manipulation of GPX4, FSP1, or an iron-handling node provides genetic support for the same mechanism. Without functional death, increased ROS, C11-BODIPY oxidation, 4-HNE or MDA accumulation, iron loading, and changes in ACSL4, SLC7A11, or GPX4 indicate redox stress or altered ferroptosis susceptibility. **Table 1** relates the three modules to their biological sequence, experimental design, and therapeutic consequences.

**Table 1:** Mechanistic structure and therapeutic implications of the three-module framework.

Module	Biological sequence	Experimental example	Therapeutic implication
Iron-flux redistribution	Heme uptake, ferritin turnover, hepcidin–FPN1 signaling, and intercellular transfer alter the recipient-cell LIP and its ferroptosis threshold.	Stable-isotope iron tracing after hepcidin–FPN1 perturbation, paired with cell-resolved LIP, ferritin turnover, phospholipid oxidation, and viability (17, 23, 24).	Tumor-directed iron loading is appropriate when labile iron accumulates in tumor cells. TAM-directed iron retention requires restriction to suppressive subsets with limited marrow and CD8+ T-cell exposure.
Lipid-hydroperoxide spectra and receptor sensing	Oxidized phospholipid species, receptor distribution, and exposure duration shape the inflammatory or suppressive response.	Oxidized-phospholipid lipidomics with receptor loss-of-function and time-resolved tumor cell–TAM co-culture across cGAS–STING, TLR, and NLRP3 routes (37, 43, 44).	Adjustment of induction intensity and timing may shorten suppressive signaling. Receptor blockade is relevant after identification of the suppressive ligand and receptor-bearing cell.
Phagocytic clearance and antioxidant buffering	Clearance transfers iron and oxidized membrane into macrophages. GPX4/FSP1 and ferritin buffering determine whether uptake contains injury or disables the phagocyte.	Live-cell efferocytosis with cell-resolved GPX4/FSP1 activity, oxidized-lipid burden, and viability after CD47–SIRPα perturbation (37, 46, 47).	Preserved clearance permits stronger tumor-directed ferroptosis. Loss of phagocyte function favors lower induction intensity or protection of the clearance-competent compartment.
Coupling across modules	The immune response shifts when oxidized-material production exceeds combined clearance and antioxidant capacity or when lipid injury reaches immune compartments.	Independent perturbation of tumor-cell GPX4 and macrophage clearance, with cell-resolved LIP, oxidized phospholipids, efferocytosis, dendritic-cell priming, and CD8+ T-cell function at matched tumor-cell death (37, 41, 59).	Escalation of tumor-cell injury remains favorable while phagocyte and T-cell functions remain intact. Extension of lipid damage into immune cells calls for lower induction intensity, receptor blockade, or immune-cell protection.

## Physiological basis of macrophage iron metabolism

Macrophages recover most of the iron required for erythropoiesis by phagocytosing senescent erythrocytes and degrading hemoglobin (11, 12). CD163 mediates uptake of hemoglobin–haptoglobin complexes, after which HO-1 releases iron from heme. Loss of HO-1 disrupts iron recycling and promotes inflammatory tissue injury (12, 19). This pathway is especially relevant in tumors with hemorrhage or vascular leakage and in macrophages positioned near these regions (20). The released iron expands the labile iron pool (LIP) and supports Fenton chemistry. Ferroptosis develops when the resulting phospholipid peroxidation causes loss of viability that is attenuated by an appropriate inhibitor (8, 10). Ferritin limits iron toxicity by sequestering iron, whereas NCOA4-mediated ferritinophagy returns ferritin iron to the LIP and increases susceptibility when antioxidant defenses are insufficient (21, 22). Hypoxia can restrain this release. In primary human macrophages, hypoxia reduced NCOA4, increased cytosolic and mitochondrial ferritin, and protected against RSL3-induced ferroptosis (23). IRP–IRE regulation further coordinates uptake, storage, and export. Consequently, similar total iron content can coexist with distinct LIPs and ferroptosis thresholds.

**Figure 2:**
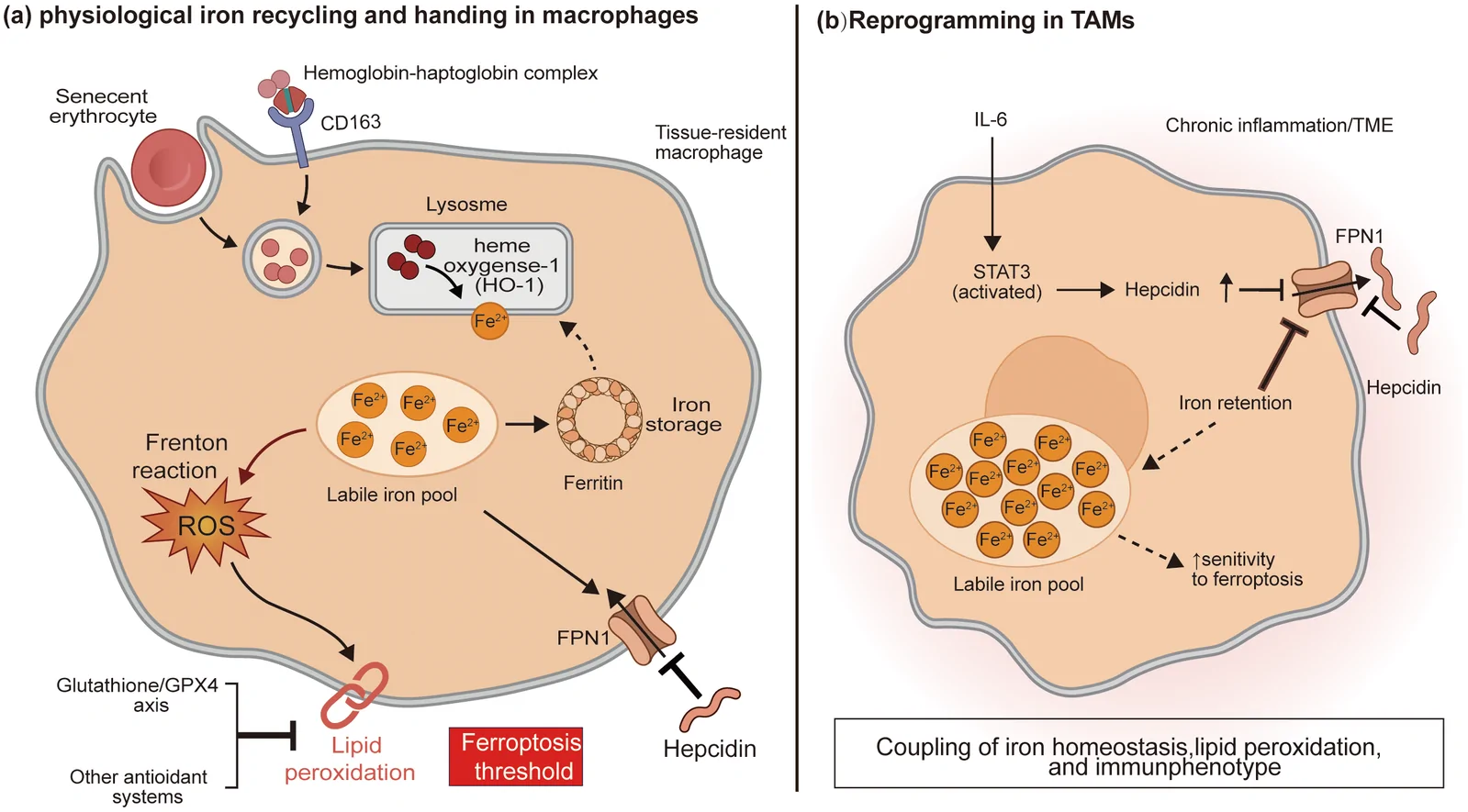
Physiological macrophage iron handling and ferroptosis susceptibility. **(a)** Macrophage iron handling: macrophages phagocytose senescent erythrocytes, internalize hemoglobin–haptoglobin through CD163, degrade heme through HO-1, store iron in ferritin, and export iron through FPN1 under hepcidin control. The LIP can support Fenton chemistry. GSH–GPX4 limits phospholipid peroxidation. **(b)** Inflammatory hepcidin signaling in TAMs: reduced FPN1-mediated export can increase iron retention. Ferritin storage and antioxidant buffering determine whether the LIP expands. TAM ferroptosis is represented by loss of viability together with iron-dependent phospholipid peroxidation that is suppressed by an appropriate inhibitor and, where feasible, reproduced by a separate genetic perturbation.

Ferroportin is the principal cellular iron exporter in mammals, and hepcidin binding promotes its internalization and degradation, thereby limiting iron release from iron-recycling macrophages (24, 25). Within tumors, hepcidin source, ferroportin abundance in malignant and myeloid cells, ferritin storage, and ongoing erythrophagocytosis shape the distribution of iron (26). These variables can generate an iron-retentive TAM that remains viable or a ferroptosis-prone TAM in which the LIP exceeds ferritin and GPX4/FSP1 capacity. The surviving TAM can continue iron sequestration, vesicle release, and clearance. By contrast, membrane lipid peroxidation in the ferroptosis-prone TAM interrupts these functions. Hepcidin–FPN1 signaling therefore changes both iron localization and the duration of macrophage function. Against this high iron flux (**Figure 2**), activation state further modifies ferroptosis susceptibility. In a macrophage culture model, iNOS-derived nitric oxide made inflammatory M1-like macrophages more resistant than M2-like macrophages by intercepting lipid-peroxidation intermediates (27). SPP1+, TREM2+, FOLR2+, and IL4I1+ TAM states add distinct lipid, heme, hypoxia, and clearance pressures that may modify this protection.

TAM heterogeneity extends well beyond the M1-like/M2-like classification. Cross-tissue single-cell analyses identify monocyte-derived and tissue-resident macrophage states marked by SPP1, TREM2, APOE/C1Q, IL4I1, and FOLR2, together with tissue-specific transcriptional programs (28). SPP1+ TAMs are enriched in hypoxic or necrotic regions and participate in extracellular-matrix remodeling and interactions with tumor cells (29). TREM2+ macrophages frequently co-express lipid-scavenging and immunoregulatory programs, and their accumulation has accompanied limited anti-PD-1 responses in the tumor systems examined (30, 31). FOLR2+ tissue-resident macrophages occupy stromal or perivascular niches associated with organized lymphoid and CD8+ T-cell responses (32). In colon cancer, IL4I1+ macrophages occupied regions of active phagocytosis, SPP1+ macrophages accumulated near hypoxia or necrosis, and FOLR2+ macrophages formed plasma-cell-associated niches (33). These spatial programs expose TAMs to different amounts of heme, lipids, dying-cell cargo, oxygen, and cytokines. Ferroptosis susceptibility rises when the influx of iron and polyunsaturated lipids exceeds ferritin storage and antioxidant protection from GPX4/FSP1 and nitric oxide (23, 27, 33). SPP1+ and TREM2+ states may approach this threshold in lipid-rich, hypoxic, or necrotic regions. Loss of FOLR2+ or IL4I1+ states may have a greater functional effect when these cells sustain organized immune niches or clear dying material (32, 33). Ontogeny establishes the initial iron-recycling and phagocytic program, spatial exposure modifies substrate load, and treatment adds an acute pulse of iron and oxidized lipids (23, 28, 33). The susceptibility of each TAM state thus reflects how its LIP and PUFA-containing phospholipid pool are balanced by ferritin storage, GPX4/FSP1 activity, and nitric oxide. The effect of cell loss depends on which phagocytic and niche functions remain in other macrophage populations. **Table 2** links these features to the expected response under ferroptotic stress.

**Table 2:** TAM states, cellular programs, responses under ferroptotic stress, and functional consequences.

TAM state	Cellular program and niche	Response under ferroptotic stress	Functional consequence
SPP1+ TAMs	Hypoxic or necrotic localization, matrix remodeling, and tumor interaction (29, 33)	Lipid and heme uptake can increase substrate load. Ferroptosis becomes more likely when this influx exceeds ferritin and GPX4/FSP1 buffering.	Selective loss may reduce suppressive matrix-remodeling functions if other phagocytes preserve clearance. Depletion of a major phagocyte population can instead increase oxidized debris.
TREM2+ APOE/C1Q TAMs	Lipid-scavenging and immunoregulatory programs associated with limited ICI response in the tumor systems examined (30, 31)	Lipid uptake can increase peroxidizable substrate while lysosomal, ferritin, and antioxidant programs increase tolerance.	Elimination may release T-cell suppression in tumors enriched for this state. Its lipid-scavenging function may also contain oxidized material.
FOLR2+ tissue-resident TAMs	Perivascular, stromal, or plasma-cell-associated niches linked to organized immune states (32, 33)	Homeostatic iron handling can favor survival. Cell loss can remove tissue support and organized immune niches.	Loss can disrupt organized lymphoid, CD8+ T-cell, or tissue-homeostatic niches.
IL4I1+ phagocytic TAMs	Enrichment in high-turnover niches and uptake of dying material (33)	Repeated uptake can increase iron and lipid load while phagocytic function remains therapeutically valuable.	Survival supports clearance and antigen handling. Ferroptotic loss can permit oxidized material to persist.
M1-like and M2-like activation programs	Broad activation programs used in culture and selected tumor studies	iNOS-derived nitric oxide increased resistance in one inflammatory macrophage model (27). Tumor-derived states also vary in ferritinophagy, lipid uptake, and antioxidant capacity.	Nitric oxide, ferritinophagy, lipid uptake, and phagocytic load determine susceptibility within either activation program.

## Ferroptosis-mediated remodeling of TAM phenotypes and functions

**Figure 3:**
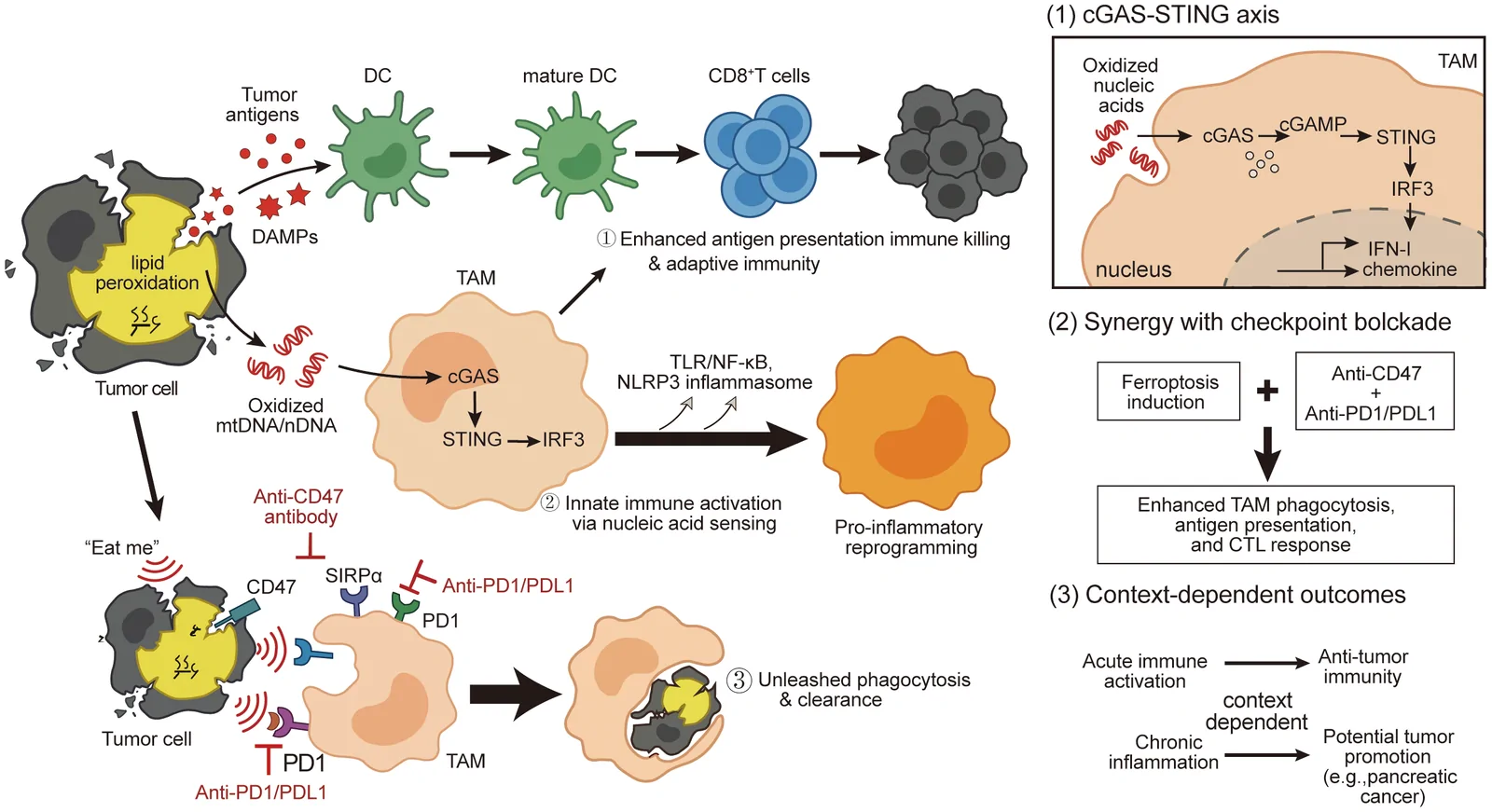
Effects of ferroptotic material on TAMs across dying-cell source, dose, timing, and clearance capacity. The diagram follows three routes: antigen and DAMP availability, innate sensing, and phagocytic handling. Antigen release can support dendritic-cell maturation and CD8+ T-cell priming. Ferroptotic-cell-derived extracellular GPX4 can impair antigen presentation. When ferroptotic death supplies a ligand for cGAS–STING, TLR, NF-κB, or NLRP3 signaling, limiting that death lowers the signal and loss of the relevant receptor or adaptor interrupts transmission. CD47–SIRPα or PD-1/PD-L1 modulation can enhance clearance through immune effects that extend beyond ferroptosis. Dying-cell source, treatment schedule, and myeloid state determine the route that dominates.

The response of TAMs to ferroptotic material depends on the source and stage of cell death, the oxidized-lipid species released, the available nucleic-acid or protein danger signals, and local phagocytic capacity (**Figure 3**) (18). Ferroptosis within TAMs removes a cellular function, whereas exposure of viable TAMs to ferroptotic tumor cells can reprogram that function. Selective loss of a suppressive macrophage state may remove trophic and antioxidant support. Conversely, loss of macrophages that mediate efferocytosis, antigen handling, or iron sequestration can weaken antitumor immunity. The effect of TAM-intrinsic ferroptosis is therefore shaped by macrophage state, spatial niche, induction intensity, treatment timing, and the function removed.

In engineered hepatocellular carcinoma models and biomimetic delivery systems, concurrent ferroptotic injury in tumor cells and M2-like or suppressive macrophages accompanied improved tumor control (34, 35). This effect is consistent with removal of trophic and antioxidant support while other macrophages retain phagocytic and antigen-presenting functions. An ovarian cancer model similarly linked macrophage ferroptosis to remodeling of the immune microenvironment (36). By contrast, phospholipid peroxidation can impair macrophage uptake of ferroptotic cells and allow oxidized debris to accumulate (37). The balance may also change over time. Early selective loss of suppressive TAMs can reduce trophic support and create space for inflammatory monocytes. As death extends across macrophage states, total uptake capacity falls, extracellular lipid exposure persists, and antigen handling declines. Recruited monocytes may then restore clearance or re-establish suppression according to the cytokine and lipid composition of the depleted niche (34, 37). Treatment outcome thus reflects which TAM state is depleted, how quickly it is replaced, and whether clearance remains sufficient. Benefit is most likely when suppressive TAMs are removed while antigen presentation, CD8+ T-cell fitness, and clearance are preserved. Damage to tissue-resident or phagocytic TAMs can reverse this balance by increasing residual oxidized material and reducing antigen handling (37–39).

Ferroptotic cancer cells have elicited immunogenic, neutral, or suppressive effects across treatment schedules and cellular compositions. Early ferroptotic cells produced vaccine-like protection in a head and neck squamous cell carcinoma model (40), whereas ferroptotic cells impaired dendritic-cell antigen presentation in another system (41). Extracellular GPX4 binding to ZP3 on dendritic cells provides a defined route through which ferroptotic material can suppress dendritic-cell metabolism and T-cell priming (42). These findings place DAMP release, dendritic-cell maturation, antigen presentation, and T-cell infiltration along a sequence in which disruption at any stage can alter the final response. Ferroptosis-associated DNA stress has also engaged cGAS–STING signaling in colorectal, pancreatic, and liver models (43–45). Mitochondrial injury, nuclear leakage, and secondary necrosis may supply different nucleic-acid ligands. Acute STING activation can support type I interferon signaling, while persistent activation can sustain tumor-promoting inflammation. Accordingly, ligand source, receptor-bearing cell, stage of death, and concurrent treatment injury determine which route operates. When ferroptotic death supplies the ligand, its attenuation should reduce the signal. Loss of the relevant TLR, NLRP3 component, or downstream adaptor should then interrupt signal transmission.

CD47–SIRPα blockade increases phagocytosis and T-cell priming through pathways that extend beyond ferroptosis (46, 47). Within ferroptosis-inducing combinations, its effect on oxidized material depends on whether enhanced uptake preserves or damages the phagocyte. Increased uptake initially restricts extracellular debris, but each engulfed cell also transfers membrane PUFAs, heme iron, and antigen into the macrophage. As this burden accumulates, lipid repair and iron storage may become insufficient, converting a clearance-promoting intervention into phagocyte dysfunction (37). Clearance efficiency, antigen presentation, and CD8+ T-cell function consequently form a connected biological sequence. Ferroptosis can modify immunity through altered antigen or danger-signal availability, innate sensing shaped by ligand identity and exposure duration, and phagocytic handling of oxidized material. The final response reflects the relation between lipid-peroxide production and the combined clearance and antioxidant capacity of the responding myeloid compartment.

## Reciprocal shaping of tumor-cell ferroptosis by TAMs

**Figure 4:**
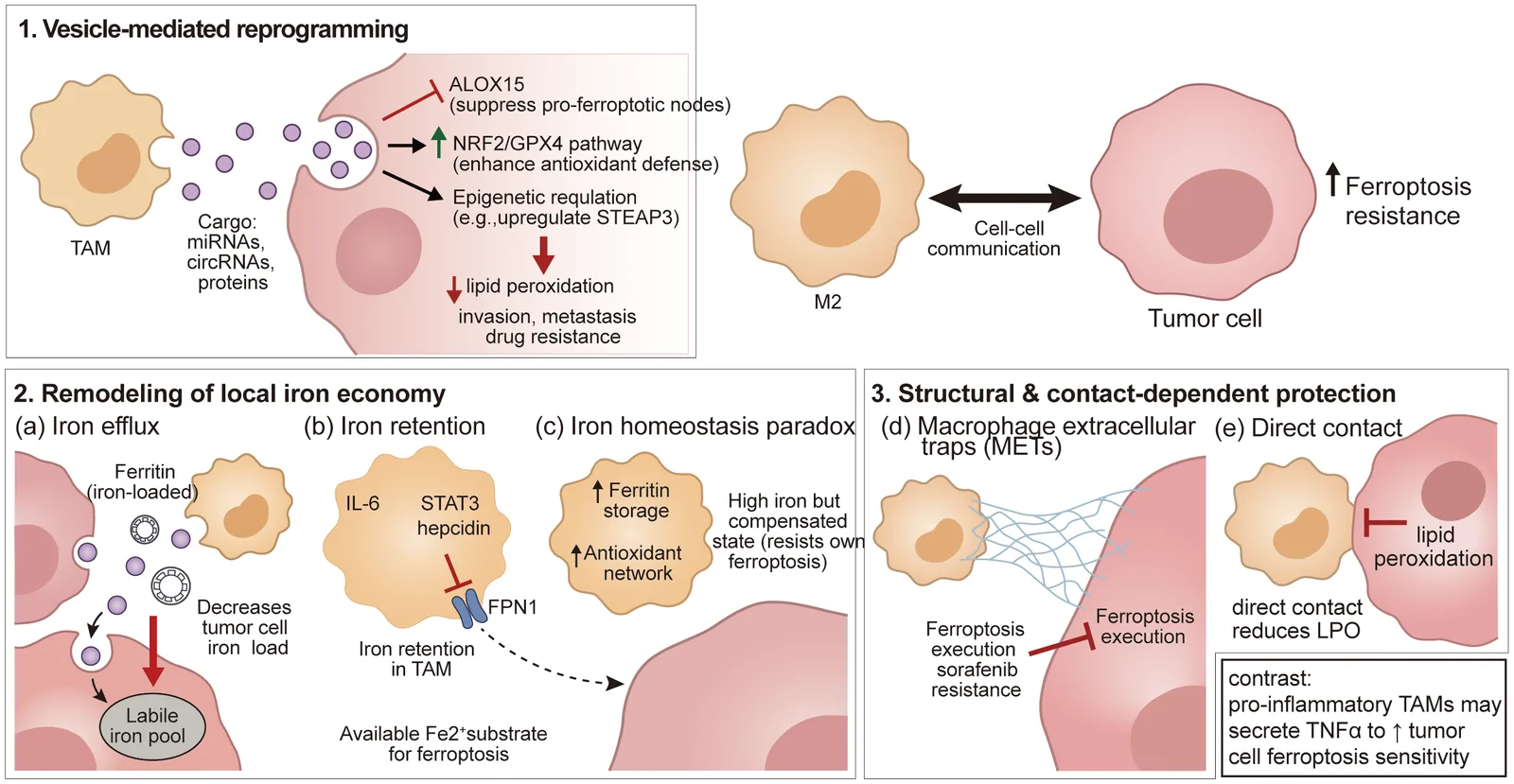
Tumor-, cargo-, and recipient-cell-specific mechanisms by which TAMs alter tumor-cell ferroptosis susceptibility. Three routes link TAMs to tumor-cell ferroptosis. Extracellular-vesicle cargo can alter ALOX15, NRF2, GPX4, STEAP3, or related pathways. Ferritin transfer, ferritinophagy, FPN1, and hepcidin can redistribute iron between donor and recipient compartments. Extracellular traps or direct contact can modify treatment-associated lipid peroxidation. Tumor type, donor and recipient cells, treatment, and antioxidant capacity determine the direction of each route. Vesicle cargo changes the recipient antioxidant pathway. Iron transfer changes the recipient LIP. Extracellular structures change the duration of treatment exposure.

TAMs modify tumor-cell ferroptosis susceptibility through extracellular-vesicle cargo, redistribution of iron-related substrates, and extracellular structures or cell contact (**Figure 4**) (15–17). Vesicle-mediated routes link donor-cell state to the ferroptosis threshold of the recipient cell. In cervical cancer, IL-4/IL-13-polarized macrophages released miR-660-5p-containing exosomes that reduced ALOX15 and tumor-cell ferroptosis (48). In cutaneous squamous cell carcinoma, macrophage-derived circ_0088494 increased STEAP3 expression and reduced erastin-associated ferroptosis (49). ANXA3-rich TAM exosomes were associated with ferroptosis suppression and lymphatic metastasis in laryngeal squamous cell carcinoma (15), while APOC1-containing macrophage exosomes altered ferroptosis resistance in osteosarcoma (50). PRDX6- and ceruloplasmin-related vesicle routes have been described in additional tumor systems (16, 17). Despite their different cargoes, these studies converge on recipient pathways controlling lipid oxidation, antioxidant capacity, or iron handling. Extension to another tumor depends on the same cargo being present and the corresponding recipient pathway remaining active.

Macrophages also alter the distribution of iron-related substrates. In glioblastoma, TAMs promoted exosomal export of ferritin-bound iron from tumor cells, lowering intracellular iron and sensitivity to GPX4 inhibition (51). Transfer of ceruloplasmin mRNA from macrophages to tumor cells provides another intercellular route of ferroptosis resistance (17). At tissue level, hepcidin–ferroportin signaling can retain iron in macrophages (24, 25), while the tumor-cell LIP remains responsive to ferritin turnover, tumor-cell ferroportin, and material transferred from macrophages (17, 20, 22). Macrophage iron retention modifies tumor-cell ferroptosis when it changes the recipient LIP or antioxidant capacity. Therapeutic stress adds extracellular structures to this exchange. In hepatocellular carcinoma, sorafenib induced macrophage extracellular traps through an ARHGDIG–IL-4–PADI4 axis, and disruption of these traps restored tumor-cell ferroptosis and improved response (52). Macrophages also protected neighboring cells in co-culture (39). Although the transferred materials differ, they converge on the recipient LIP, antioxidant capacity, or duration of lipid peroxidation. Ferritin-bound iron export reduces substrate for lipid oxidation. Ceruloplasmin transfer and macrophage contact strengthen antioxidant protection, whereas extracellular traps can prolong a protective cytokine interface during treatment. A TAM may therefore shield tumor cells through several routes that depend on its state and treatment exposure.

## Tumor microenvironmental conditions that modulate ferroptosis–TAM interactions

Iron availability supplies the substrate for ferroptosis, but its cellular distribution and antioxidant capacity determine which compartment is damaged. Local hemorrhage and erythrocyte turnover increase heme uptake by macrophages. Ferritin storage and ferroportin-mediated export then regulate entry of this iron into the labile pool (11, 12, 20). Hepcidin-driven ferroportin loss favors macrophage iron retention (24, 25). Macrophage ferroptosis becomes more likely as the LIP expands beyond ferritin storage and GPX4/FSP1 capacity, whereas hypoxic suppression of ferritinophagy can limit this expansion (23, 24). Tumor-cell susceptibility rises when ferritin turnover or intercellular transfer increases the tumor-cell LIP (17, 22). In a Kras-driven pancreatic model, iron loading or Gpx4 perturbation promoted oxidized-DNA and STING-associated inflammation and accelerated tumorigenesis (44). Hypoxia provides a second source of cell-specific variation. In primary human macrophages, hypoxia suppressed ferritinophagy and increased ferritin-dependent protection from ferroptosis (23). In another tumor system, hypoxic tumor-derived exosomes reduced macrophage inflammatory activity and ferroptosis through NDUFV2 (53). Transient acidosis in breast cancer increased tumor-cell ferroptosis and M1-like macrophage features (54). Together, oxygen tension, pH, lipid composition, and cell identity shape the redox state of the recipient compartment.

Cytokines change ferroptosis thresholds differently across tumor cells, CD8+ T cells, neutrophils, and macrophage states. IFN-γ from activated CD8+ T cells can reduce SLC3A2/SLC7A11 and increase tumor-cell ferroptosis during immunotherapy or radiotherapy (55, 56). Direct contact with cytotoxic CD8+ T cells has also been reported to sustain GPX4 downregulation and ferroptosis in melanoma cells (57). In chemotherapy-resistant breast cancer, an IL-1β-dependent CD4+ T-cell circuit promoted neutrophil ferroptosis and release of immunosuppressive mediators (58). IL-4/IL-13-polarized macrophages instead transferred anti-ferroptotic vesicle cargo to cervical cancer cells (48). Effector T cells are themselves vulnerable to lipid stress. In tumor-infiltrating CD8+ T cells, CD36-dependent fatty-acid uptake increased lipid peroxidation and ferroptosis and reduced antitumor activity in mouse models, with corresponding associations in human tumors (59). The effect of low cystine depends on extracellular cystine, fatty-acid uptake, mitochondrial stress, and antioxidant capacity in the affected cell. Together, these findings support a possible relay in which IFN-γ sensitizes tumor cells, oxidized material subsequently enters macrophages and granulocytes, and suppressive lipids feed back on CD8+ T cells (55, 58, 59). This feedback narrows the treatment window as viability and cytotoxicity decline in the effector population that initiates tumor sensitization. Extracellular vesicles add a parallel route by transferring proteins or RNAs that modify ALOX15, NRF2, GPX4, ceruloplasmin, or iron handling in recipient tumor cells (15–17). The direction of this route follows vesicle source, cargo composition, recipient cell, and tumor lineage. In both relays, a signal generated by one cell changes the ferroptosis threshold of the next.

## Effects of ferroptosis–TAM interactions on the tumor microenvironment

**Figure 5:**
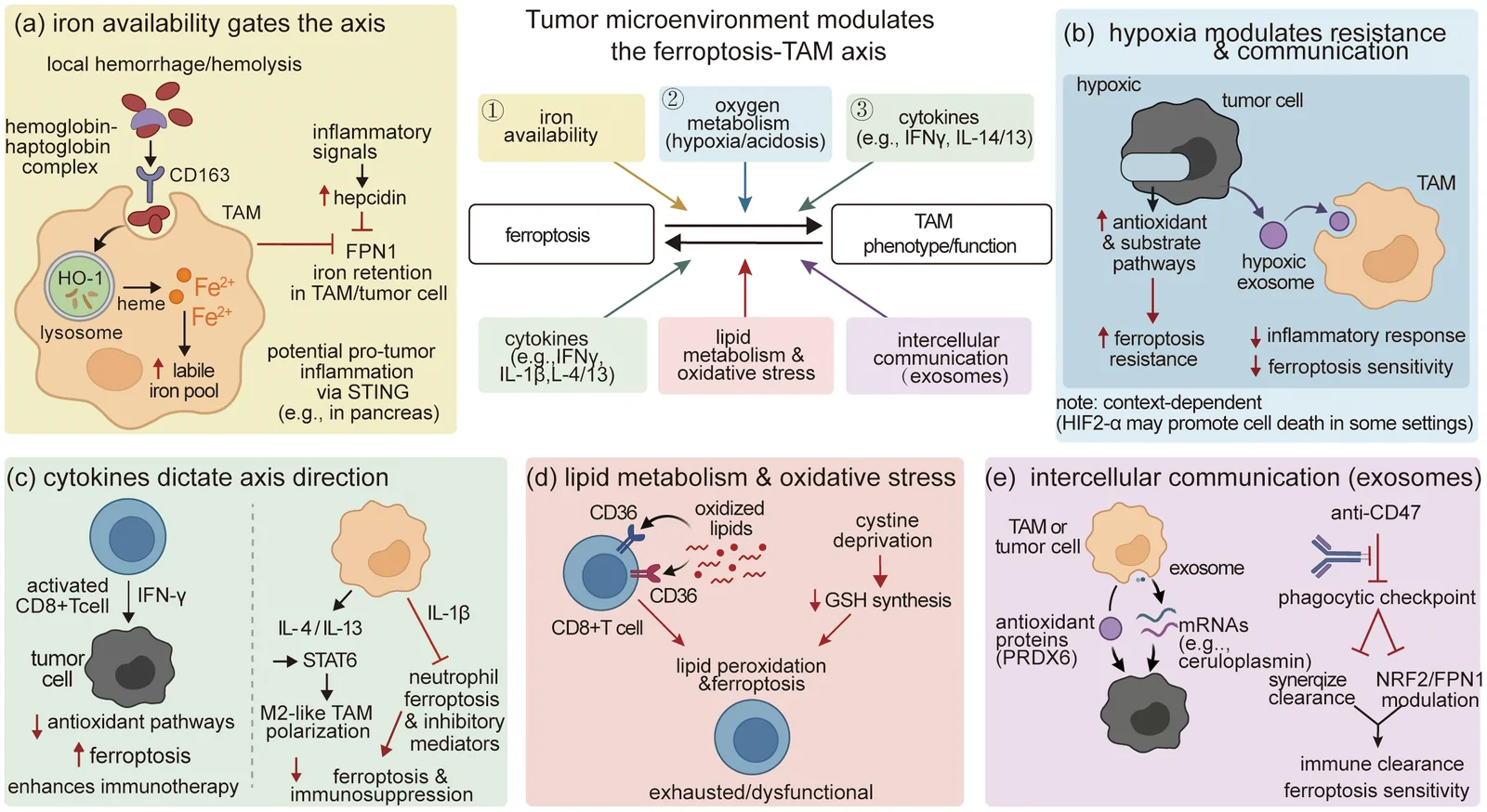
Tumor-microenvironmental modulation of the ferroptosis–TAM axis by oxygen tension, pH, cytokines, lipid supply, and vesicle cargo. The schematic depicts iron availability, oxygen and pH, cytokines, lipid metabolism, and extracellular vesicles as inputs to the ferroptosis–TAM axis. Iron and hepcidin–FPN1 signaling alter substrate distribution. Ferroptotic death combines phospholipid peroxidation, loss of viability, and suppression by an appropriate ferroptosis inhibitor. Hypoxia, acidosis, IFN-γ, IL-1β, IL-4/IL-13, CD36-dependent lipid uptake, and vesicle cargo act in different cell types and tumor systems. Donor cell, transferred material, recipient pathway, treatment, and exposure time determine whether the signal increases or decreases ferroptosis. **(a)** Iron availability gates the axis. **(b)** Hypoxia modulates ferroptosis resistance and intercellular communication. **(c)** Cytokines determine the direction of the axis. **(d)** Lipid metabolism and oxidative stress regulate ferroptosis susceptibility. **(e)** Exosomes mediate intercellular communication.

Ferroptotic death and concurrent tissue injury redistribute iron and oxidized lipids within the TME (**Figure 5**). In Kras-driven pancreatic cancer, a high-iron diet or Gpx4 perturbation increased oxidized nucleosides, STING-associated macrophage infiltration, and tumorigenesis, whereas ferroptosis inhibition or macrophage depletion attenuated these effects (44). This model links oxidized nucleosides to STING-associated inflammation after high-iron feeding or Gpx4 perturbation. Granulocytic ferroptosis provides a second route to immune suppression. In tumor-bearing mice, ferroptotic PMN-MDSCs released oxygenated lipids that impaired T-cell activity, and ferroptosis inhibition reduced suppression and improved checkpoint blockade (60). In chemotherapy-resistant breast cancer, ferroptotic neutrophils released PGE2, IDO-associated mediators, and oxidized lipids that impaired CD8+ T-cell proliferation and cytotoxicity (58). Despite different tumor and treatment settings, both granulocyte routes converge on lipid-mediated T-cell dysfunction. Phagocytic handling provides another control point. Macrophage phospholipid peroxidation can impair uptake of ferroptotic cells, while defined oxidized phospholipids can serve as uptake cues through receptors such as TLR2 (37). Changes in the mechanical properties of ferroptotic cells may also modify efferocytosis (61). Oxidized phosphatidylcholine, hydroperoxyeicosatetraenoic acid-containing phospholipids, and 4-HNE adducts engage different receptors and uptake pathways. Their abundance and receptor distribution shape whether phagocytosis is maintained or T-cell function is suppressed.

Metabolic conditions modify ferroptosis susceptibility and macrophage behavior through distinct intracellular pathways. In colorectal cancer, histone lactylation increased IGF2BP2–NRF2-associated resistance to ferroptosis (62). In hepatocellular carcinoma, a FOXM1–IGF2BP3–RRM2 pathway linked ferroptosis resistance to M2-like macrophage polarization (63). Hypoxic tumor-derived vesicles suppressed macrophage inflammation and ferroptosis through NDUFV2 (53), whereas transient acidosis activated a ZFAND5–SLC3A2 pathway, increased breast cancer ferroptosis, and elicited M1-like macrophage features (54). Although these routes use different intracellular nodes, each changes substrate supply or antioxidant capacity in tumor cells or macrophages. Metabolic competition extends this effect across compartments. Glutamine synthetase activity supports an immunosuppressive macrophage program, whereas its inhibition promotes inflammatory macrophage features and limits metastasis (64). In head and neck squamous cell carcinoma, glutamine inhibition combined with CD47 blockade increased radiotherapy-associated ferroptosis (65). Itaconate uptake through SLC13A3 can confer ferroptosis resistance and impair tumor immunity (66). Cystine availability similarly controls GSH synthesis, with its contribution varying across cell types and competing metabolic demands.

## Cell-specific immune consequences and possible IDO1–kynurenine–AhR control of TAM ferroptosis

Indoleamine 2,3-dioxygenase 1 (IDO1) and tryptophan 2,3-dioxygenase 2 (TDO2) convert tryptophan to kynurenine. Kynurenine activates the aryl hydrocarbon receptor (AhR) and contributes to T-cell dysfunction, regulatory programs, and checkpoint resistance (67, 68). Tumor-cell studies suggest two routes through which the same pathway could alter TAM-intrinsic ferroptosis. IDO1–AhR signaling increased NRF2-dependent pentose phosphate pathway activity and ferroptosis resistance in lung cancer (69). In another cancer-cell system, 3-hydroxykynurenine inhibited NCOA4 and ferritinophagy (70). The first route increases NADPH-dependent antioxidant buffering, whereas the second limits ferritin-derived iron release. Together, they suggest that IDO1–AhR activity could protect TAMs by increasing antioxidant capacity or retaining iron in ferritin. This effect may be most relevant in lipid-laden or iron-loaded macrophage states. Kynurenine add-back after IDO1 loss would test whether kynurenine mediates the effect, while NCOA4 restoration would distinguish iron retention from NRF2-dependent antioxidant buffering.

Any TAM-intrinsic IDO1–AhR effect would occur within a TME in which ferroptosis has opposing cell-specific consequences. IFN-γ from activated CD8+ T cells can lower tumor-cell SLC3A2/SLC7A11, and fatty-acid remodeling can increase ACSL4-dependent tumor-cell ferroptosis (55, 71). Within the same effector population, CD36-dependent lipid uptake can drive T-cell ferroptosis and reduce cytotoxicity (59). Ferroptotic PMN-MDSCs and neutrophils can release oxidized lipids and other suppressive mediators (58, 60). Regulatory T cells (Tregs) depend on GPX4 and FSP1 to preserve redox control and suppressive function (72, 73). The immune effect of material released by ferroptotic tumor cells also depends on ligand identity and receptor expression. HMGB1–AGER signaling promoted macrophage inflammation in one system (74). In separate models, ferroptotic cells impaired dendritic-cell priming, and extracellular GPX4–ZP3 signaling defined one route to dendritic-cell suppression (41, 42). These cell-specific responses mean that the same tumor-cell injury can strengthen one immune compartment while weakening another.

**Figure 6:**
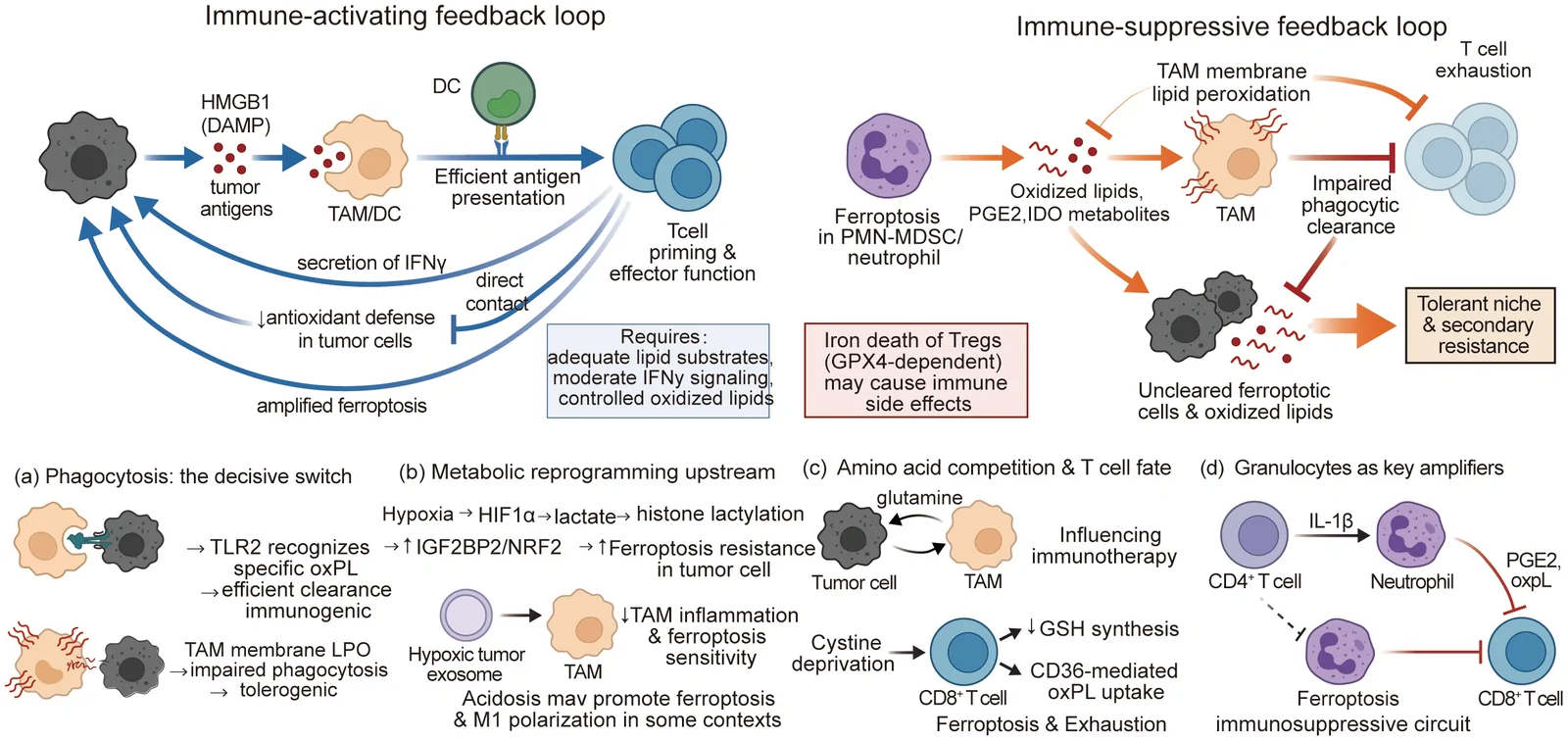
Immune outcomes of ferroptosis–TAM feedback networks. This figure connects the cell-specific routes described in the text. In an immune-activating configuration, tumor-cell ferroptosis can increase antigen or DAMP availability while phagocytic clearance, dendritic-cell function, and CD8+ T-cell fitness remain intact. In an immune-suppressive configuration, granulocytic ferroptosis or persistent oxidized lipids can impair T cells and macrophage clearance. IFN-γ, CD36, TLR2, cGAS–STING, hypoxia, acidosis, and extracellular vesicles act according to cell type, tissue, dose, and exposure duration. Iron location sets the initially injured compartment. Lipid species and receptor expression set signal reception. Clearance and antioxidant capacity set the spread of injury. The upper portion contrasts immune-activating and immune-suppressive feedback loops. **(a)** Phagocytosis shapes the balance between clearance and tolerance. **(b)** Metabolic reprogramming alters TAM inflammation and ferroptosis susceptibility. **(c)** Amino acid competition links tumor cells and TAMs to CD8-positive T-cell fate. **(d)** Granulocytes amplify immune suppression.

Macrophages control whether these cell-specific effects remain local or spread through tissue. Their susceptibility varies with iNOS-derived nitric oxide, oxygen tension, ferritinophagy, the LIP, and antioxidant capacity across defined macrophage states (23, 27, 38). Extracellular vesicles further redistribute iron-related cargo between cells (75). SPP1+ and TREM2+ states may accumulate lipids in hypoxic or necrotic niches, while FOLR2+ and IL4I1+ states can sustain organized immune niches or active clearance (28, 33). These properties create two feedback loops. Tumor-cell ferroptosis can increase antigen or danger-signal availability while macrophage and dendritic-cell functions remain intact. T-cell activation can then reinforce tumor-cell ferroptosis through IFN-γ and ACSL4 (55, 71). In the opposing loop, granulocytic ferroptosis releases oxygenated lipids and macrophage lipid peroxidation reduces clearance (37, 58, 60). Macrophage replacement adds a slower component. After TAM depletion, recruited monocytes may restore uptake capacity and then acquire suppressive or inflammatory programs according to local cytokines, lipids, and oxygen. The same initial loss can consequently produce transient immune relief followed by renewed suppression, or sustained clearance with inflammatory repopulation. Both outcomes depend on whether oxidized-material production remains within the combined clearance and antioxidant capacity of nearby myeloid and dendritic cells. Below this threshold, antigens and danger signals are processed while phagocytes remain functional. Above it, impaired macrophage uptake, extracellular GPX4, and granulocytic lipids reduce antigen presentation and T-cell function (37, 41, 42). Iron location, lipid species, receptor use, and residual phagocytic capacity define the network shown in **Figure 6**.

## Intervention strategies targeting the ferroptosis–TAM axis

Therapeutic strategies along the ferroptosis–TAM axis induce ferroptosis in tumor cells or selected suppressive TAMs, or disrupt macrophage-mediated protection of tumor cells. Dual induction and macrophage reprogramming reduced tumor growth in hepatocellular carcinoma, biomimetic-delivery, and gastric cancer models (34, 35, 76). Vesicle cargo and iron handling provide additional targets in specific tumors (15–17). For spatially controlled delivery, selectivity depends on biodistribution and cell-level uptake (77, 78). Hepcidin inhibition or ferroportin stabilization is most likely to benefit an iron-retentive state in which the recipient LIP rises (24, 25). System xc− or GPX4 inhibition remains a common experimental route to ferroptosis, although available compounds also affect other cellular processes. Concordant pharmacological attenuation and genetic target perturbation link these interventions to ferroptotic death (6, 8, 10). Checkpoint blockade or macrophage reprogramming has increased tumor control in selected preclinical systems (79, 80). In hepatocellular carcinoma, concurrent ferroptosis targeting in tumor cells and macrophages and disruption of macrophage extracellular traps improved sorafenib-based treatment (34, 52). The module order suggests a treatment sequence. Localization of the rising LIP and lipid hydroperoxides identifies the initial target. Preservation or restoration of clearance in that niche then permits stronger tumor-directed injury. Extension of lipid damage into immune cells calls for lower induction intensity, receptor blockade, or antioxidant protection of the affected immune compartment.

Nanodelivery platforms combine iron delivery, GSH depletion, GPX4 inhibition, ROS generation, phototherapy, or immunomodulatory cargo (81–83). Their biological sequence begins with tumor-directed oxidative stress, which increases the lipid and debris load entering TAMs. Macrophage scavenging then determines whether injury remains local or reaches dendritic cells and T cells. However, nanoparticle uptake by TAMs can reduce delivery to tumor cells while increasing macrophage iron and lipid load. A macrophage-rich tumor may therefore convert high tumor accumulation into myeloid sequestration. Delayed or stimulus-responsive cargo release can shift exposure from TAMs that internalize the carrier to neighboring tumor cells. Therapeutic selectivity depends on tumor accumulation, cell-level exposure, macrophage phagocytosis, and immune-cell injury. Endogenous macrophage vesicles have delivered anti-ferroptotic cargo in cervical, laryngeal, fibrosarcoma, and other tumor models (15–17). Engineered M1 exosomes and exosome-like vesicles have delivered pro-ferroptotic or immunomodulatory cargo (77, 84). Macrophage-membrane camouflage may improve tumor accumulation, although carrier composition can also modify inflammation and cargo delivery (78). Manufacturing consistency, scalability, and immunogenicity remain barriers to repeated systemic use, and clinical efficacy remains unknown (10).

## Clinical relevance and therapeutic outlook

Clinical evidence remains limited. The CNSI-Fe study evaluated the safety and feasibility of intratumoral CNSI-Fe in 19 patients with advanced solid tumors (85). The cohort had no concurrent control, and some outcomes were recorded after study treatment had been discontinued. The effects of subsequent therapy, patient selection, and the natural course of disease therefore cannot be separated from those of CNSI-Fe. Overall, ferroptosis-directed cancer therapy remains at an early stage of clinical translation.

Erastin- and RSL3-like compounds remain experimental because of pharmacokinetic, selectivity, and safety limitations (10). Sorafenib, temozolomide, sulfasalazine-related system xc− modulators, and haloperidol affect several cellular pathways, so their clinical activity cannot be attributed to ferroptosis alone. For iron-containing or ROS-generating nanomedicines, paired biopsies could localize iron delivery and phospholipid oxidation in tumor cells while tracking CD8+ T-cell function, dendritic-cell activity, and macrophage uptake (37, 41, 59). Nanoplatform studies also inform tumor localization and dose control (86, 87). Single-gene and pan-cancer signatures correlate with macrophage infiltration, checkpoint expression, and prognosis in selected cohorts (88–90). These associations vary with tumor composition, proliferation, hypoxia, and treatment exposure. A practical selection profile combines tumor-cell LIP and GPX4/FSP1 status, spatial TAM composition, and baseline oxidative injury in CD8+ T cells and phagocytes. Early on-treatment biopsies could then show whether tumor-cell phospholipid oxidation increases while immune-cell function is preserved. Imaging probes, multiplex tissue analysis, and lipidomics can help localize this change to the target compartment.

The safety window is narrow because off-target lipid peroxidation can impair antitumor T cells and normal tissues (59, 72). Tumor-targeted delivery, dose control, and reversible activation can reduce this exposure (86, 87). Checkpoint blockade, CD47–SIRPα blockade, innate immune agonists, and vesicle cargo also modify immunity through routes independent of ferroptosis. Tumor cells remain the initial target in many solid tumors. Subset-selective TAM targeting is appropriate when suppressive function and ferroptosis susceptibility coincide in a spatially accessible population and other macrophages preserve clearance and tissue homeostasis (32, 34, 37). Systemic iron indices remain relevant because macrophages control a large fraction of whole-body iron recycling (11, 25). Organoid–immune co-cultures, macrophage–tumor co-cultures, and humanized mouse models reproduce complementary aspects of stromal and myeloid biology (91–93). Patient selection then follows the compartment at greatest risk. Tumor-dominant vulnerability supports tumor-directed induction, whereas susceptibility concentrated in suppressive TAMs supports subset-selective targeting with preserved clearance. Baseline oxidative injury in CD8+ T cells or phagocytes instead argues for immune-cell protection and lower systemic exposure. Paired tissue and blood sampling in early-phase trials can connect target engagement with immune-cell function and systemic iron handling (**Table 3**).

**Table 3:** Cell-type-specific determinants of ferroptosis susceptibility and therapeutic implications.

Cell population	Cellular features in tumor systems	Dominant determinants	Therapeutic implication
Tumor cells	Ferroptosis sensitivity varies with tumor lineage, PUFA-phospholipid pools, LIP, GPX4/FSP1 activity, hypoxia, and treatment stress (6, 55).	PUFA-phospholipid pools, LIP, GPX4/FSP1, hypoxia, and treatment stress	Tumor cells remain the initial target in many solid tumors, with immune-cell fitness setting the upper dose limit (6, 55).
Inflammatory TAM states	iNOS increased resistance in inflammatory macrophages. Hypoxia reduced ferritinophagy and protected macrophages from RSL3-induced ferroptosis (23, 27, 38).	Nitric oxide, iron recycling, GPX4/FSP1, phagocytic load, and cytokine exposure	Preservation is favored when these cells support clearance, antigen handling, or inflammatory signaling.
Lipid-laden or suppressive TAM states	Concurrent ferroptosis in tumor cells and suppressive macrophages improved tumor control in hepatocellular carcinoma, ovarian cancer, and biomimetic-delivery systems (34–36).	Lipid uptake, ferritinophagy, CD163/HO-1, NRF2/GPX4, and hypoxia	Subset-selective targeting is appropriate when other macrophages retain clearance and antigen-handling capacity.
Tissue-resident TAMs	FOLR2+ and other tissue-resident states occupy organ-specific and spatially organized immune niches (28, 32).	Developmental origin, heme exposure, homeostatic phagocytosis, and local cytokines	Their homeostatic and organized immune functions narrow the therapeutic window for depletion.
Monocyte-derived TAMs	Recruitment signals, hypoxia, lactate, lipid uptake, and local differentiation generate distinct monocyte-derived states (28).	Recruitment signals, hypoxia, lactate, lipid uptake, and local differentiation	The local state and niche guide the choice between reprogramming and selective ferroptosis.
CD8+ T cells	CD36-dependent lipid uptake drove ferroptosis and reduced cytotoxicity in mouse tumors. Corresponding lipid and functional changes were associated in human tumors (55, 59).	Fatty-acid uptake, lipid peroxidation, and antioxidant capacity	Protection of CD8+ T cells preserves the effector arm during tumor-cell ferroptosis (55, 59).
Neutrophils and PMN-MDSCs	Ferroptotic PMN-MDSCs in tumor-bearing mice and neutrophils in chemotherapy-resistant breast cancer released lipids and mediators that suppressed T-cell function (58, 60).	Oxidized phospholipids, PGE2 and IDO-associated mediators, and inflammatory stress	Restricted granulocytic injury limits release of T-cell-suppressive lipid species.
Tregs	GPX4 and FSP1 maintain redox control and suppressive function in Tregs (72, 73).	GPX4-dependent redox control and local lipid availability	Local Treg injury may reduce suppression, while systemic lipid injury can also reach effector T cells.

## Solid tumors versus hematologic malignancies

Most evidence on ferroptosis–TAM interactions comes from solid tumors, where hypoxia, necrosis, hemorrhage, extracellular-matrix barriers, and spatially segregated myeloid niches shape local iron and lipid exposure. By contrast, hematologic malignancies develop in marrow, blood, and lymphoid ecosystems where malignant cells coexist with erythroblastic islands, iron-recycling macrophages, stromal cells, and therapy-sensitive progenitors (94, 95). Transfusional iron, ineffective erythropoiesis, systemic inflammation, and treatment-related cytopenias can distribute ferroptotic pressure across these compartments. Spatial targeting in solid tumors may concentrate injury in tumor cells or suppressive TAM niches. Systemic exposure in hematologic malignancies can also damage marrow macrophages that support erythropoiesis, antigen handling, and hematopoietic recovery. Shared iron-handling programs between malignant myeloid cells and macrophages further complicate cell-of-origin assignment. Consequently, malignant-cell death, macrophage function, progenitor injury, and hematopoietic recovery become linked efficacy and safety outcomes in marrow. **Table 4** summarizes the corresponding differences in dose, tissue sampling, and safety.

**Table 4:** Key differences between ferroptosis-macrophage interactions in solid tumors and hematologic malignancies.

Feature	Solid tumors	Hematologic malignancies	Therapeutic consequence
Tissue architecture	Spatially bounded lesions with hypoxic, necrotic, hemorrhagic, and invasive niches	Diffuse marrow, blood, and lymphoid compartments with continuous cell trafficking	Local delivery can restrict exposure in solid tumors. Hematologic malignancies distribute exposure across compartments.
Dominant iron setting	Intratumoral hemorrhage, vascular leakage, necrosis, heme uptake, and local ferritin or ferroportin handling (11, 20)	Erythrophagocytosis, ineffective erythropoiesis, transfusions, and systemic iron redistribution (11, 94, 95)	Cell-resolved tumor and macrophage iron links local injury to systemic or marrow iron burden.
Macrophage role	Clearance, antigen presentation, matrix remodeling, angiogenesis, and local immune suppression	Support of erythroblastic islands, hematopoiesis, marrow recovery, and immune regulation	Macrophage death removes different tissue functions in the two settings.
Therapeutic risk	Injury to T cells and clearance-competent TAMs	Additional injury to progenitors, erythropoiesis, and post-treatment marrow recovery	Marrow exposure narrows the dose window and extends safety assessment to hematopoietic recovery.
Treatment exposure	Local or intratumoral delivery can concentrate ferroptotic pressure within a lesion	Systemic treatment exposes malignant cells, marrow macrophages, and hematopoietic progenitors together (94, 95)	Cellular co-exposure narrows treatment selectivity in hematologic malignancies.

## Discussion

The immune consequence of ferroptosis emerges from exchanges among dying cells, the TAM compartment, and neighboring immune populations. Cell-autonomous models locate ferroptosis execution and defense nodes, whereas immune models describe downstream activation or suppression (7, 8, 18). The three modules bridge these levels by following intercellular transfer. Iron redistribution identifies the first injured compartment and the amount of iron available for release. Lipid species and receptor-bearing cells translate this injury into a recipient-cell response. Clearance and antioxidant buffering then govern the duration and tissue spread of oxidized material. In turn, phagocytosis transfers iron and oxidized membranes into macrophages, feeding back into iron storage and lipid sensing. When debris production exceeds macrophage uptake and redox capacity, local tumor-cell death can therefore progress to broader immune-cell damage. This reciprocal coupling distinguishes the three-module framework from a cell-autonomous ferroptosis map or a final classification of immune outcome.

The iron-flux module separates tissue iron abundance from the reactive iron available to individual cells. Macrophages can carry a large iron burden while ferritin storage, nitric oxide, hypoxia-mediated suppression of ferritinophagy, and parallel antioxidant defenses keep the LIP below the death threshold (11, 12, 23). Hepcidin-mediated ferroportin loss retains iron in macrophages, while adjacent tumor-cell iron remains responsive to ferritin turnover, tumor-cell ferroportin, and intercellular transfer (17, 24, 25). Ferroptotic pressure consequently follows the recipient LIP relative to its antioxidant capacity. An iron-mediated route begins with the source and donor compartment, passes through ferritin turnover or ferroportin, and ends in the recipient LIP. Serum ferritin and bulk tumor iron describe systemic or tissue burden, whereas cell-resolved LIP identifies the compartment exposed to ferroptotic pressure.

The sensing module follows the molecular species released by injured cells and the receptors that receive them. Oxidized phospholipids, HMGB1, DNA, and extracellular GPX4 engage different receptors on macrophages and dendritic cells (37, 42, 74). cGAS–STING signaling in colorectal, pancreatic, and liver models also changes with exposure duration (43–45). Acute activation can support type I interferon responses, whereas persistent DNA stress can sustain tumor-promoting inflammation. This temporal divergence depends on the nucleic-acid pool, receptor-bearing cell, stage of death, and concurrent treatment injury. In systems where ferroptotic death supplies the initiating ligand, attenuation of that death should lower the signal, and loss of the receptor or adaptor should interrupt transmission. The same sequence can connect defined oxidized lipids to TLR or NLRP3 signaling.

The clearance-buffering module governs whether oxidized material is removed or accumulates. Macrophage lipid peroxidation can reduce efferocytosis, and ferroptotic tumor cells can impair dendritic-cell priming (37, 41). Macrophages can also protect stressed tumor cells through direct contact or vesicle cargo (15, 16, 39). Clearance and antioxidant buffering operate in sequence. Removal of irreversibly dying cells limits extracellular oxidized debris, while protection of a recoverable tumor cell preserves disease. Phagocytosis, however, transfers heme iron and oxidized membrane lipids into the macrophage. Repeated uptake increases the macrophage LIP and lipid burden, progressively consuming ferritin storage and GPX4/FSP1 buffering. The macrophage initially contains the injury. Once uptake and redox capacity decline together, residual oxidized material persists and reaches dendritic cells and T cells (23, 37, 41). This progression links clearance back to iron redistribution and lipid sensing. The outcome reflects target-cell fate, phagocyte function, cell-resolved iron, and the amount of oxidized material that remains.

Cell identity shapes how the three modules combine. Tumor-cell ferroptosis can cooperate with CD8+ T-cell IFN-γ (55), whereas CD36-dependent ferroptosis within the T cell removes the effector arm (59). Ferroptosis of a suppressive TAM may be beneficial when clearance-competent macrophages remain. Loss of a FOLR2+ organized immune niche or a phagocytic macrophage can instead increase the functional cost of treatment (32, 37). SPP1+, TREM2+, FOLR2+, and IL4I1+ states differ in spatial niche, lipid handling, and phagocytic demand (28, 33). Subset identity establishes the starting conditions, and subsequent exposure to heme, hypoxia, lipids, cytokines, and dying cells shifts the balance between substrate load and redox defense. SPP1+ and TREM2+ states may become vulnerable when lipid and iron influx exceeds GPX4/FSP1 and ferritin capacity (23, 28, 33). Depletion of FOLR2+ and IL4I1+ states may remove organized immune niches or clearance functions (32, 33). Therapeutic outcome therefore combines susceptibility of the targeted subset with the macrophage function that is lost.

Solid tumors and hematologic malignancies place the same molecular target within different tissue architectures. Spatially bounded lesions may permit local dosing around necrotic or suppressive niches. In marrow, however, systemic treatment reaches iron-recycling macrophages, erythroid precursors, malignant myeloid cells, and progenitors required for recovery (94, 95). The therapeutic window consequently depends on both tissue architecture and molecular target. Depletion of a suppressive TAM in a solid tumor can translate into loss of erythropoietic support or impaired marrow recovery after systemic exposure. Spatial target engagement guides dosing in solid tumors, whereas progenitor viability, erythropoiesis, and hematopoietic recovery constrain dosing in marrow.

The 19-patient CNSI-Fe phase 1 cohort provides early information on the feasibility and safety of intratumoral delivery (85). The study had no concurrent control, and some outcomes were recorded after treatment ended. Effects of subsequent therapy, patient selection, and the natural course of disease cannot be separated from CNSI-Fe exposure. The cohort therefore does not establish ferroptosis-specific clinical efficacy. Paired tissue and blood samples in future trials could localize iron and phospholipid oxidation in tumor cells and identify concurrent injury to CD8+ T cells or phagocytes. Comparative efficacy could then be interpreted alongside the identity of the injured cell compartment.

Two complementary factorial designs can test coupling among the three modules. The first independently perturbs tumor-cell GPX4 and macrophage clearance, then measures cell-resolved LIP, oxidized phospholipids, efferocytosis, dendritic-cell priming, and CD8+ T-cell function at matched tumor-cell death. If clearance sets the propagation threshold, equal tumor-cell death should produce different immune responses when clearance remains intact or becomes saturated. The second design alters hepcidin–FPN1 signaling while holding the ferroptosis trigger constant. If iron redistribution sets the first injured compartment, a shift in the recipient LIP should relocate the injury. Repeating both designs in a second tumor type would show how tissue architecture and treatment schedule modify each route.

## Conclusions

Ferroptosis alters tumor immunity through the movement of iron and oxidized material among malignant cells, TAMs, and neighboring immune populations. TAMs occupy a central position because they redistribute iron, clear dying cells, and buffer lipid damage. When ferroptotic injury remains concentrated in tumor cells and phagocytic and dendritic-cell functions are preserved, antigen handling and antitumor immunity may be maintained. As lipid damage reaches CD8+ T cells or granulocytes, TAM redox capacity declines, or debris exceeds clearance, the same process can favor immune suppression. The three-module framework connects these outcomes to iron redistribution, molecular sensing, and clearance. It also separates tumor-directed induction, selective depletion of suppressive TAMs, and protection of phagocytic macrophages into distinct delivery and dosing strategies. Clinical development should combine target-cell exposure with spatial pharmacodynamics and early assessment of immune-cell function. Current clinical observations remain exploratory, and prospective cell-resolved studies will determine whether these relationships predict treatment response across tumor types.

## Glossary

4-HNE: 4-hydroxynonenal
ACSL4: acyl-CoA synthetase long-chain family member 4
AhR: aryl hydrocarbon receptor
ALOX15: arachidonate 15-lipoxygenase
AGER: advanced glycosylation end-product specific receptor
CD36: cluster of differentiation 36
CD47: cluster of differentiation 47
CD163: cluster of differentiation 163
CD8+ T cells: cluster of differentiation 8-positive T lymphocytes
circRNA: circular RNA
CNSI-Fe: carbon nanoparticle-loaded iron
CoQ10: coenzyme Q10
cGAS–STING: cyclic GMP–AMP synthase–stimulator of interferon genes pathway
CTLA-4: cytotoxic T-lymphocyte-associated protein 4
CTLs: cytotoxic T lymphocytes
DCs: dendritic cells
DAMPs: damage-associated molecular patterns
EVs: extracellular vesicles
FPN1: ferroportin 1
FSP1: ferroptosis suppressor protein 1
GPX4: glutathione peroxidase 4
GSH: glutathione
HMGB1: high mobility group box 1
HIF-1α: hypoxia-inducible factor 1 alpha
HO-1: heme oxygenase 1
HPETE: hydroperoxyeicosatetraenoic acid
IDO: indoleamine 2,3-dioxygenase
IFN-γ: interferon gamma
IGF2BP2: insulin-like growth factor 2 mRNA-binding protein 2
IL: interleukin
ICIs: immune checkpoint inhibitors
iNOS: inducible nitric oxide synthase
IRE: iron-responsive element
IRP: iron regulatory protein
LAG-3: lymphocyte activation gene 3
LIP: labile iron pool
MDA: malondialdehyde
MDSCs: myeloid-derived suppressor cells
miRNA: microRNA
mtDNA: mitochondrial DNA
NF-κB: nuclear factor kappa B
NLRP3: NOD-like receptor family pyrin domain containing 3
NRF2: nuclear factor erythroid 2–related factor 2
NCOA4: nuclear receptor coactivator 4
PGE2: prostaglandin E2
PD-1: programmed cell death protein 1
PD-L1: programmed death-ligand 1
PMN-MDSCs: polymorphonuclear myeloid-derived suppressor cells
PUFAs: polyunsaturated fatty acids
ROS: reactive oxygen species
SIRPα: signal regulatory protein alpha
SLC3A2: solute carrier family 3 member 2
STAT3: signal transducer and activator of transcription 3
TAMs: tumor-associated macrophages
TANs: tumor-associated neutrophils
TDO2: tryptophan 2,3-dioxygenase
TLR: toll-like receptor
TME: tumor microenvironment
Tregs: regulatory T cells
ZP3: zona pellucida glycoprotein 3
